# The Detrimental Effect of Machiavellian Leadership on Employees’ Emotional Exhaustion: Organizational Cynicism as a Mediator

**DOI:** 10.5964/ejop.v11i4.988

**Published:** 2015-11-27

**Authors:** Panagiotis Gkorezis, Eugenia Petridou, Theodora Krouklidou

**Affiliations:** a Department of Economics, Aristotle University of Thessaloniki, Thessaloniki, Greece; Aalborg University, Aalborg, Denmark

**Keywords:** Machiavellian leadership, organizational cynicism, emotional exhaustion, mediation

## Abstract

Numerous empirical studies have examined predictors of emotional exhaustion. In this vein, both positive and negative leadership styles have been associated with this outcome. Yet, little is known about the role of Machiavellian leadership in fostering employees’ emotional exhaustion. As such, we investigated the relationship between Machiavellian leadership and emotional exhaustion. Even more, we investigated an explanatory mechanism of this association by encompassing organizational cynicism as a mediator. Results showed that Machiavellian leadership has a both direct and indirect, through organizational cynicism, on employees’ emotional exhaustion.

Emotional exhaustion, described as “feelings of being emotionally drained by one’s work” (Bakker & Costa, 2014, p. 2), constitutes one of the core dimensions of burnout. In fact, scholars (Cropanzano, Rupp, & Byrne, 2003; Maslach, Schaufeli, & Leiter, 2001) have argued that it is the most important part of the specific phenomenon. Accordingly, empirical research has shown that emotional exhaustion exerts stronger and more consistent effects on important outcomes compared to the other two components of burnout, namely disengagement and personal accomplishment (Demerouti, Bakker, Nachreiner, & Schaufeli, 2001; Lee & Ashforth, 1996).

Given its distinct and salient influence on key outcomes such as organizational citizenship behavior (Cropanzano et al., 2003), job satisfaction (Karatepe & Tekinkus, 2006; Mulki, Jaramillo, & Locander, 2006), organizational commitment (Bozionelos & Kiamou, 2008) and job performance (Halbesleben & Bowler, 2007; Janssen, Lam, & Huang, 2010; Wright & Cropanzano, 1998), numerous empirical studies have examined its antecedents. In this vein, the literature has addressed the role of both positive and negative leadership style (e.g., Thomas & Lankau, 2009; Wu & Hu, 2009). Regarding the latter, though, little attention has been given to the relationship between Machiavellianism and emotional exhaustion.

Machiavellianism has again received attention after the considerable stream of research in the 1970s and 1980s (Dahling, Whitaker, & Levy, 2009). This is largely attributed to the recent corporate scandals and numerous cases of misconduct and malfeasance in organizations which have prompted researchers to invigorate their interest in the dark side of organizations (Belschak, Den Hartog, & Kalshoven, 2015). Given that Machiavellian leaders have its roots in this dark side and are perceived as opportunists, manipulators and cheaters (Gunnthorsdottir, McCabe, & Smith, 2002; Sakalaki, Richardson, & Thépaut, 2007) this leadership style became a topic of interest for leadership and management literature.

Thus, the purpose of the present paper is to examine the impact of Machiavellian leadership on employees’ emotional exhaustion. More importantly, we attempt to explain why this relationship occurs by providing an important explanatory mechanism. To this end, we highlight organizational cynicism, that is a negative attitude toward one’s employing organization resulting from the perception that organization lacks integrity (Dean, Brandes, & Dharwadkar, 1998), as a mediator that accounts for the effect of Machiavellianism on emotional exhaustion (Figure 1).

**Figure 1:**
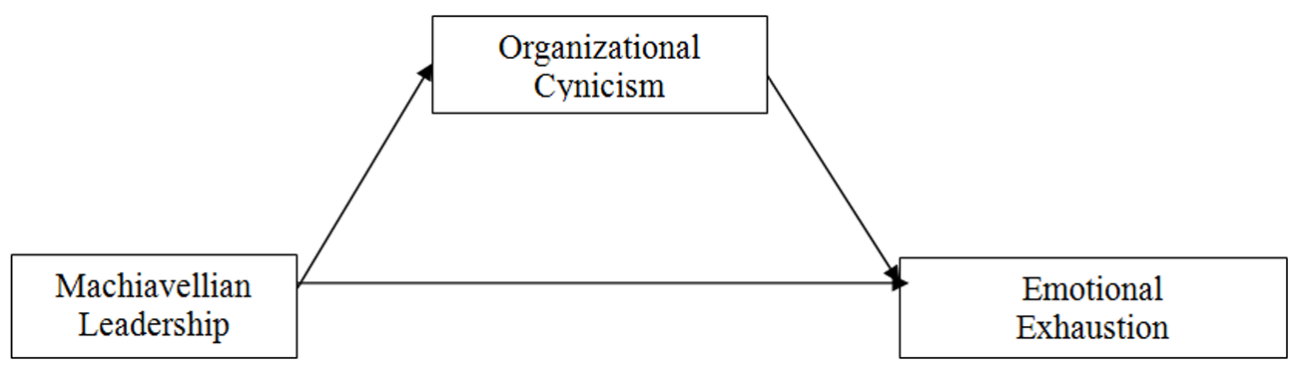
The hypothesized model.

## Literature Review

### Machiavellianism

The construct of Machiavellianism has emerged in the literature after the work of Christie and Geis who described high Machs as those that “manipulate more, win more, are persuaded less, persuade others more, and otherwise differ significantly from their low Machiavellian counterparts” (Christie & Geis, 1970, p. 312). Drawing on the seminal books of Machiavelli *The Prince* (1513/1981) and *The Discourses* (1531/1984), they recognized some common aspects of Machiavellians such as “willingness to utilize manipulative tactics and act amorally and endorse a cynical, untrustworthy view of human nature” (Dahling et al., 2009, p. 220).

In the organizational domain, prior studies have shown that Machiavellianism is related to a plethora of outcomes including lower organizational citizenship behavior, decreased job satisfaction, higher turnover and increased counterproductive work behavior (Dahling et al., 2009; Fehr, Samson, & Paulhus, 1992; O’Boyle, 2012; Sakalaki et al., 2007; Wilson, Near, & Miller, 1996). Furthermore, extensive research has examined the relationship between Machiavellianism and work performance providing, though, inconsistent results. For example, several studies have reported a positive association between Machiavellianism and performance (e.g., Dahling et al., 2009), others have shown a negative effect of Machiavellianism on performance (Gable & Topol, 1988), whereas a last stream of research has demonstrated a non-significant relationship (Gable & Topol, 1991; Hunt & Chonko, 1984). Despite the extant research on Machiavellianism, less is known in the realm of leadership (Dahling et al., 2009). We attempt to contribute to the Machiavellian leadership literature by investigating the effect of leader Machiavellianism on employees’ emotional exhaustion through the mediation of organizational cynicism.

### Machiavellian Leadership and Emotional Exhaustion

There is an increasing interest among scholars regarding the role of leadership in affecting followers’ mood and emotions (Bono, Foldes, Vinson, & Muros, 2007; Gooty, Connelly, Griffith, & Gupta, 2010). Both positive and negative leader behaviors are likely to elicit favorable and unfavorable emotional reactions. For example, scholars have focused on the pivotal role that leaders play in mitigating or enhancing employees’ emotional exhaustion. More specifically, prior studies have demonstrated that positive leadership such as leader-member exchange (Becker, Halbesleben, & O’Hair, 2005; Thomas & Lankau, 2009), authentic (Spence Laschinger & Fida, 2014) and transformational (Seltzer, Nomerof, & Bass, 1989) may affect such employees’ feelings. By contrast, substantial empirical research has revealed that abusive leadership (Aryee, Sun, Chen, & Debrah, 2008; Tepper, 2007; Wu & Hu, 2009) increases employees’ emotional exhaustion (Chi & Liang, 2013; Wu & Hu, 2009). Given that leader Machiavellianism is related to employees’ perceptions of abusive supervision (Kiazad, Restubog, Zagenczyk, Kiewitz, & Tang, 2010) we expect that the former will also augment employees’ feelings of emotional exhaustion. In addition, Drory and Gluskinos (1980) have argued that Machiavellian leaders show little concern for both their interpersonal relationships with their followers and their feelings. This may be attributed to the fact that high Machs are self-interested, solely focus on their achievements (Cooper & Peterson, 1980; Sakalaki et al., 2007) as well as they exhibit low empathy for others (Bagozzi et al., 2013; Paal & Bereczkei, 2007). Thus, following the above logic we postulate that Machiavellian leaders will enhance employees’ emotional exhaustion.

H1: Machiavellian leadership is positively related to employees’ emotional exhaustion.

### Machiavellian Leadership and Organizational Cynicism

Although organizational cynicism has emerged many years ago, there is relatively insufficient empirical research. Similar to Machiavellian construct, organizational cynicism has recently attracted increasing attention due to the corporate scandals and the unethical behavior of leaders which have augmented employees’ cynicism toward the organization (Bommer, Rich, & Rubin, 2005; Naus, van Iterson, & Roe, 2007). In addition to its pertinence to contemporary business environment, organizational cynicism has been a topic of concern because it has been negatively related to core outcomes, such as job satisfaction, organizational commitment and job performance (e.g. Chiaburu, Peng, Oh, Banks, & Lomeli, 2013).

In their recent meta-analysis, Chiaburu and his colleagues (2013) have attempted to accumulate some core antecedents of organizational cynicism. More importantly, they argued that employees’ cynical attitudes towards the organization may be determined by organizational factors that manifest lack of integrity. Such distrust and lack of both integrity and morality may arise when leaders demonstrate Machiavellian behavior because high Mach individuals exhibit elevated levels of increased narcissism (McHoskey, 1995), mistrust and cynicism (McHoskey & Hicks, 1999). On a related note, Machiavellian individuals have been described as having the tendency to cheat and lie (Lee & Ashton, 2005; Ross & Robertson, 2000). Brown and Treviño (2006, p. 604) noted that “Machiavellian leaders are motivated to manipulate others in order to accomplish their own goals. They have little trust in people and in turn, tend not to be trusted by others”. Moreover, scholars argued that high Machs “disregard standards of morality and see value in behaviors that benefit the self at the expense of others” (Dahling et al., 2009, p. 228).

Given that supervisor-subordinate relationship is one of the most influential in the workplace (Sluss & Ashforth, 2008) and the former serves as a representative of the organization constituting the lens whereby subordinates perceive the organization (Lord, Brown, & Freiberg, 1999), we posit that leader Machiavellianism may be related to organizational cynicism. Hence, based on the aforementioned theoretical and empirical arguments we propose that Machiavellian leaders will enhance employees’ organizational cynicism.

H2: Machiavellian leadership is positively related to employees’ organizational cynicism.

### Machiavellian Leadership, Organizational Cynicism and Emotional Exhaustion

According to the above arguments, we have suggested that Machiavellian leadership predicts both emotional exhaustion (Hypothesis 1) and organizational cynicism (Hypothesis 2). In parallel with this, scholars have demonstrated that organizational cynicism is positively related to emotional exhaustion (Johnson & O’Leary-Kelly, 2003). More specifically, they have argued that the fatigue and a broad host of negative reactions that emanates from organizational cynicism may result in producing enhanced levels of personal strain and emotional exhaustion. Furthermore, as noted above, leaders are considered to be representatives of each organization. As a result, employees’ perceptions about the leader are likely to affect their attitudes about the organization. In this regard, Machiavellian leaders who are perceived as unethical and of low integrity may lead employees to be skeptical and negative towards the organization which in turn is may result in increased emotional exhaustion.

Hence, in line with previous research that highlights the mediating role of organizational cynicism (Evans, Goodman, & Davis, 2010; Johnson & O’Leary-Kelly, 2003) and taking into consideration the aforementioned hypotheses and the relationship between organizational cynicism and emotional exhaustion, we propose that organizational cynicism will act as a partial mediator between Machiavellian leadership and employees’ emotional exhaustion.

H3: Organizational cynicism partially mediates the relationship between Machiavellian leadership and employees’ emotional exhaustion.

## Method

### Sample and Procedure

We collected data from employees working in a Greek private hospital. One of the authors contacted human resource department which in turn administered the questionnaires. Overall, 150 questionnaires were allocated to participants and 122 were returned producing a response rate of 80 per cent. Among these, 40.2 per cent of respondents were male and 68.9 per cent was less than 45 years old. Furthermore, a great percentage of the sample (63.9 per cent) has been employed in a permanent basis. Last, the majority of the respondents has worked for the present organization for less than 10 years (56.6 per cent) and has work experience less than 20 years (78.7 per cent).

### Measures

Machiavellian leadership and organizational cynicism used a five-point Likert scale where 1=strongly disagree and 5=strongly agree. Likewise, emotional exhaustion was measured using a five-point scale ranging from 1 (never) to every day (5). All scale reliabilities (Machiavellian leadership: α = 0.94; organizational cynicism: α = 0.89; emotional exhaustion: α = 0.89) were acceptable, exceeding the value (.70) suggested by Nunnally, Bernstein, and Berge (1967).

#### Machiavellian leadership

We assessed Machiavellian leader behavior adapting the ten-item scale from Allsopp, Eysenck, and Eysenck (1991). An example item is “My supervisor often acts in a cunning way in order to get what he wants”.

#### Organizational cynicism

We measured organizational cynicism using the four items developed by Brandes, Dharwadkar, and Dean (1999) (as cited in Kim, Bateman, Gilbreath, & Andersson, 2009). An example item for this scale is “I believe top management says one thing and does another”.

#### Emotional exhaustion

We assessed emotional exhaustion using five items from Maslach Burnout Inventory (Maslach, Jackson, & Leiter, 1996). A sample item includes “I feel emotionally drained from my work”.

#### Control variables

We controlled for five demographic characteristics, namely gender, age, employment status, job tenure, organizational and job tenure. Given that the bivariate correlations between control variables and outcomes were not significant we excluded them from our regression analyses (Becker, 2005).

### Confirmatory Factor Analysis

We conducted confirmatory factor analyses (using AMOS 20) in order to examine the discriminant validity of the constructs. The results (Table 1) showed that our three-factor baseline model provided the best fit to the data (x^2^(145) = 258.68, *p* < .01, CFI = .94, TLI = .93, IFI = .94, RMSEA = 0.08). Moreover, we employed Harman’s single factor test (Podsakoff, MacKenzie, Lee, & Podsakoff, 2003) in order to examine the magnitude of common method bias. The results indicated a poor fit for the one factor model (x^2^ [148] = 658.36, *p* < .01, CFI = .72, TLI = .68, IFI = .72, RMSEA = 0.17).

## Results

Means, standard deviations and correlations of the present variables are shown in Table 2. Machiavellian leadership is positively related to both organizational cynicism (*r* = .56, *p* < .01) and emotional exhaustion (*r* = .51, *p* < .01). Also, organizational cynicism is related with emotional exhaustion (*r* = .49, *p* < .01).

**Table 1:** Confirmatory Factor Analysis.

Model	X^2^	*df*	Δχ^2^	CFI	TLI	IFI	RMSEA
*Three factor model*	258.68	145		.94	.93	.94	.08
Two factor model: Machiavellian leadership and organizational cynicism	460.99	147	202.31**	.83	.80	.83	.13
Two factor model: Machiavellian leadership and emotional exhaustion	472.38	147	213.70**	.82	.79	.82	.14
Two factor model: Organizational cynicism and emotional exhaustion	471.34	147	212.66**	.82	.79	.82	.14
One factor model	658.36	148	399.68**	.72	.68	.72	.17

*p ≤ .05. **p ≤ .01.

**Table 2:** Descriptive Statistics and Correlations.

Variable	*M*	*SD*	1	2	3	4	5	6	7
1. Gender	0.60	0.49							
2. Age	2.08	0.93	-.18*						
3. Employment status	1.46	0.69	-.16	-.35**					
4. Job tenure	2.65	0.95	-.13	.77**	-.35**				
5. Organizational tenure	2.99	1.06	-.05	.58**	-.43**	.74**			
6. Machiavellian leadership	2.60	1.16	-.16	.12	-.17	.06	.07		
7. Organizational cynicism	2.79	1.17	-.13	.03	.03	-.07	-.08	.56**	
8. Emotional exhaustion	2.85	0.97	-.08	.17	-.17	.10	.17	.51**	.49**

*p ≤ .05. **p ≤ .01.

In order to test our hypotheses we used the three-step approach suggested by Baron and Kenny (1986). According to this process, mediation is supported when the following three conditions occur: a) the independent variable relates to the dependent variable b) the independent variable relates to the mediating variable and c) the mediating variable relates to the dependent variable and the relationship of the independent variable with the dependent variable is significantly lower in magnitude (or insignificant) in the third equation than in the second. In order to provide further support for the mediation hypothesis, we also conducted bootstrap analysis -1000 bootstrap samples with 95% confidence intervals - in SPSS using macro developed by Preacher and Hayes (2004). This approach has the advantage of not assuming normality of sampling distribution.

The results (Table 3) showed that Machiavellian leadership significantly relates to both emotional exhaustion (β = .51, *p* < .01) and organizational cynicism (β = .56, *p* < .01) supporting, thus, our first two hypotheses and, accordingly, the two conditions of the mediation hypothesis. Moreover, the present results demonstrated that organizational cynicism is related to emotional exhaustion (β = .30, *p* < .01) and the relationship between Machiavellian leadership and emotional exhaustion is lower (β = .34, *p* < .01) when we added organizational cynicism in the equation. Thus, we found support for the third condition and, therefore, our third hypothesis. Likewise, the results from bootstrapping analysis corroborated our findings as the bias corrected confidence interval of the specific indirect effect did not contain zero (ranging between .07 and .24). Taken together, the results showed that organizational cynicism partially mediates the relationship between Machiavellian leadership and emotional exhaustion.

**Table 3:** Regression Results for Testing Hypotheses

Variable	Organization Cynicism	Emotional Exhaustion	
		Step 1	Step 2
**Machiavellian leadership**	.56**	.51**	.34**
**Organizational cynicism**	-	-	.30**
**Adjusted R^2^**	.31**	.25**	.31**

*p ≤ .05. **p ≤ .01.

In addition, as already mentioned we excluded our control variables from our regression analyses. However, in order to provide more robust results, we conducted the same process encompassing our control variables. The results also supported our hypotheses indicating not only the positive relationship between Machiavellian leadership and both emotional exhaustion (β = .49, *p* < .01) and organizational cynicism (β = .57, *p* < .01) but also the indirect effect of Machiavellian leadership on emotional exhaustion through organizational cynicism (bias corrected confidence interval ranged between .08 and .25).

## Discussion

Burnout and, in particular, emotional exhaustion has important negative consequences for both employees and organizations (e.g., Cropanzano et al., 2003; Wright & Cropanzano, 1998). As such, in their attempt to investigate its antecedents, scholars have investigated the role of negative leadership styles in generating emotional exhaustion (Chi & Liang, 2013; Wu & Hu, 2009). Yet, no prior study, to the best of authors’ knowledge, has examined the relationship between Machiavellian leadership and this outcome. Using a sample of hospital employees, we found that Machiavellian leaders play an important role in enhancing emotional exhaustion. Thus, we contribute to the extant substantial literature on emotional exhaustion which addresses its antecedents (e.g. Grandey, 2003; Houkes, Janssen, de Jonge, & Bakker, 2003; Stordeur, D'hoore, & Vandenberghe, 2001). Moreover, our findings extend recent empirical research (Den Hartog & Belschak, 2012; Kiazad et al., 2010; O’Boyle, 2012; Zettler & Solga, 2013) on Machiavellian leadership by demonstrating emotional exhaustion as a negative outcome.

In addition, the present study aims to provide a better understanding of the mechanisms underlying this effect by incorporating organizational cynicism. Congruent with prior studies that point to the mediating role of organizational cynicism (Evans, Goodman, & Davis, 2010; Johnson & O’Leary-Kelly, 2003) our findings suggest that this construct partially mediates the relationship between leader Machiavellianism and employees’ emotional exhaustion. Relatedly, our results also add to the emerging organizational cynicism literature and corroborate previous studies that have highlighted the vital role of leadership in affecting such this attitudinal outcome. Nevertheless, although these studies have largely addressed the relationship between positive styles of leadership and organizational cynicism (Cole, 2006; Gkorezis, Petridou, & Xanthiakos, 2014) our present findings highlight the deleterious effect of negative leadership and in particular Machiavellianism. Last, the present results are in congruence with prior findings that have found the relationship between organizational cynicism and emotional exhaustion (Johnson & O’Leary-Kelly, 2003).

### Practical Implications

Our present results have some useful implications for organizations. The findings indicated that Machiavellian leaders have a detrimental impact on employees’ organizational cynicism and emotional exhaustion. Given that both outcomes negatively affect core attitudinal and behavioral outcomes such as job satisfaction, organizational commitment, intention to quit and job performance (e.g., Chiaburu et al., 2013; Cropanzano et al., 2003) it is of utmost importance that organizations should avoid recruiting and nourishing Machiavellian leadership.

Moreover, organizations should identify and pay attention when such leadership behaviors are manifested. To achieve this, organizations may cultivate an open and friendly communication and culture which will allow subordinates to somehow express their complaints and worries regarding their high Mach supervisor in the top management. Even more, in the case of Machiavellian leadership, organizations could benefit from making the specific leaders aware of their inclinations and, consequently, their harmful effect on employees’ outcomes (Bagozzi et al., 2013).

Taken together, at a more general level the present findings underscore the core role of integrity, authenticity and ethics *vis-a-vis* leadership and organization. Lack of these issues may lead employees to experience enhanced levels of cynicism and emotional exhaustion, which, in turn, will result in negative employee and organizational outcomes.

### Limitations and Future Research

As in any research, the present study has some limitations that need to be taken into consideration. First, we employed a cross-sectional design. As a consequence, it is difficult to examine the direction of causality. Moreover, common method variance may confound our findings since we gathered data from a single source, namely employees. Also data were drawn from the health care sector and in particular from a large Greek hospital. Thus, we should be cautious in generalizing the present results to other contexts.

Based on the above limitations, some stimulating avenues for future research may emerge. For example, future studies might use an experimental approach in order to test for the causality of the present relationships. In a similar methodological vein, further research should cope with common method bias by collecting data using either a longitudinal or a multi-source (i.e., supervisors) design. Furthermore, researchers could focus on potential moderators which may alleviate or bolster the negative influence of Machiavellian leaders on both organizational cynicism and emotional exhaustion. For example, as regards the latter, scholars (Suls & Martin, 2005) have argued that neurotic individuals exhibit more severe emotions in response to their problems in their environment. Hence, future research may examine the moderating role of followers’ levels of neuroticism in the relationship between Machiavellian leadership and emotional exhaustion. In the same mode, further research could investigate other important intervening mechanisms that may account for the impact of Machiavellian leaders on followers’ emotional exhaustion.
